# Sleep Improves Prospective Remembering by Facilitating Spontaneous-Associative Retrieval Processes

**DOI:** 10.1371/journal.pone.0077621

**Published:** 2013-10-15

**Authors:** Susanne Diekelmann, Ines Wilhelm, Ullrich Wagner, Jan Born

**Affiliations:** 1 Institute of Medical Psychology and Behavioral Neurobiology, University of Tuebingen, Tuebingen, Germany; 2 Child Development Center, University Children's Hospital Zurich, Zurich, Switzerland; 3 Division of Mind and Brain Research, Department of Psychiatry and Psychotherapy, Charite University Medicine Berlin, Berlin, Germany; University College London, United Kingdom

## Abstract

Memories are of the past but for the future, enabling individuals to implement intended plans and actions at the appropriate time. Prospective memory is the specific ability to remember and execute an intended behavior at some designated point in the future. Although sleep is well-known to benefit the consolidation of memories for past events, its role for prospective memory is still not well understood. Here, we show that sleep as compared to wakefulness after prospective memory instruction enhanced the successful execution of prospective memories two days later. We further show that sleep benefited both components of prospective memory, i.e. to remember *that* something has to be done (prospective component) and to remember what has to be done (retrospective component). Finally, sleep enhanced prospective remembering particularly when attentional resources were reduced during task execution, suggesting that subjects after sleep were able to recruit additional spontaneous-associative retrieval processes to remember intentions successfully. Our findings indicate that sleep supports the maintenance of prospective memory over time by strengthening intentional memory representations, thus favoring the spontaneous retrieval of the intended action at the appropriate time.

## Introduction

The main function of memories of the past is to regulate future behavior. Experimental evidence for the driving role of intentions in maintaining memories was first provided in 1927 by Bluma Zeigarnik [1], after her professor, Gestalt psychologist Kurt Lewin, noticed that waiters had good recollection of still unpaid orders, but forgot them rapidly after the bill had been paid. In this sense, memories are motivated and intended as they are basically formed for predicting future events and planning behavior, which has received little attention in memory research ever since [2]. Prospective memory is the prototype of future-directed memory as it directly refers to the ability to form an intention to do something in the future and to remember to realize the intended action when a specific cue is encountered in the environment (cue-based) [3]. Prospective memory is ubiquitous in human everyday activities: Whether to take a cake out of the oven or to publish a scientific paper, all our plans heavily depend on the ability to maintain intentions in memory and to retrieve them after a shorter or longer time interval [4,5]. 

Prospective memory comprises two distinct sub-processes, the prospective component, which refers to the ability to remember *that* something has to be done (intent), and the retrospective component, which describes the ability to remember what has to be done (content) [5,6]. There is evidence suggesting that both components can vary independently [6–8]. Moreover, on the neurobiological level, the prospective component appears to rely predominantly on activity in prefrontal cortex regions [9,10], whereas medial-temporal lobe structures, including hippocampus, are involved in recall of the retrospective component of prospective memory [11]. Supporting this view, patients with lesions to the prefrontal cortex fail to remember *that* they were supposed to do something but successfully remember the content of the intention when prompted, whereas patients with damage to the medial-temporal lobe typically remember *that* they were supposed to do something but are not able to retrieve *what* it was they were about to do [11]. 

However, evidence for the two sub-components of prospective memory differentially relying on these specific brain regions is not unequivocal because, depending on the processes primarily used for solving a prospective memory task, the prospective component can presumably be successfully accomplished by engaging both prefrontal executive functions and hippocampal memory functions [12]. Two main processes have been proposed to successfully accomplish prospective remembering: Monitoring refers to a process that keeps the intention active in mind and searches the environment for a cue to signal that the intended action can be appropriately executed [13,14]. Such monitoring is a resource-dependent attention-based process mainly recruiting prefrontal functions [13]. Prospective remembering can also take place spontaneously in an associative memory-based process when the prospective memory cue is encountered [15]. Spontaneous-associative retrieval can occur if the prospective memory cue is stored and sufficiently connected to the intended action in a hippocampal associative memory network so that encountering the cue automatically activates the associated intended action through spreading activity in the network [15,16]. According to recently proposed models, e.g. the dynamic multiprocess framework [17] and a computational model [18], spontaneous-associative retrieval and monitoring are not mutually exclusive processes but rather interact dynamically to provide optimal prospective memory performance. Whether one or the other process prevails in the execution of a certain intention is thereby assumed to depend on a variety of factors, such as characteristics of the prospective memory task, contextual details, and individual differences [12,16–18]. 

Despite the critical importance of prospective memory in everyday life, the mechanisms underlying the maintenance of prospective memories over longer time intervals are largely unknown. In recent years, there has been an upsurge of evidence that sleep plays a vital role in the process of memory formation of retrospective memories, i.e., memories of past events [19–23]. Subjects who are allowed to sleep after learning show better memory retention than subjects who spend an equivalent amount of time awake [24]. Sleep thereby actively facilitates the consolidation and reorganization of memories for long-term storage rather than merely passively protecting memories against decay and interference [25]. This active processing of memories during sleep also entails some kind of selection mechanism determining whether or not previously acquired memories will be subjected to sleep-dependent consolidation. For example, recent evidence suggests that sleep selectively consolidates memories that are relevant for future behavior, e.g., that are needed for a future memory test [26,27] or are associated with an anticipated monetary reward [28]. 

Although such findings strongly corroborate the notion that sleep is critically implicated in processes of memory retention, the role of sleep in maintaining prospective memories is not well understood. In a recent study, we tested whether sleep after forming an intention increases the likelihood of executing the intended behavior after a delay of two days. We found that compared to a wake control condition, subjects who were allowed to sleep after instructing the intention were almost twice as likely to perform the intended action successfully at retesting [29]. Likewise, Scullin and McDaniel [30] provided evidence that sleep can improve prospective memory performance after a delay of 12 hrs. In this study, subjects who obtained a night of sleep during the retention interval detected more prospective memory cues in an ongoing task at retesting than subjects who spent a day awake. Further analyses suggested that sleep might have supported spontaneous retrieval processes to improve prospective remembering [30]. 

Although these previous findings are suggestive of a memory-improving effect of sleep on prospective memories, they leave open several important questions. It remains unclear, for example, whether sleep supports prospective memory in general or whether it facilitates a particular sub-process of prospective memory, i.e. the prospective component or the retrospective component. Also the mechanisms underlying sleep’s effect on prospective memory, for instance whether sleep enables the preferential recruitment of specific processes to solve a prospective memory task, remain to be elucidated. In the present study, we addressed these questions by testing whether (i) sleep selectively benefits the prospective component, the retrospective component, or both, and (ii) whether sleep fosters prospective remembering by particularly supporting the engagement of memory-based spontaneous-associative retrieval processes. 

## Materials and Methods

### Participants

A total of 35 healthy young adults (9 females, mean age [± SD]: 23.83 ± 3.74), with regular sleep-wake cycles (≥ 6 hours sleep per night) and no shift work for at least six weeks prior to the experiments participated in the study. (Note: Another set of data obtained from the same group of subjects is published in [29].) Subjects reported no history of any neurological, psychiatric or endocrine disorder and did not take any medication at the time of the experiments. Ingestion of caffeine and alcohol was not allowed from the day before until the end of the experiments. Prior to the experimental night, subjects in the sleep group spent one adaptation night in the sleep laboratory. All subjects gave written informed consent and were paid for participation. The study was approved by the local ethics committee of the University of Luebeck, Germany. All experiments were conducted according to the principles expressed in the Declaration of Helsinki.

### Prospective memory task

**Figure 1:**
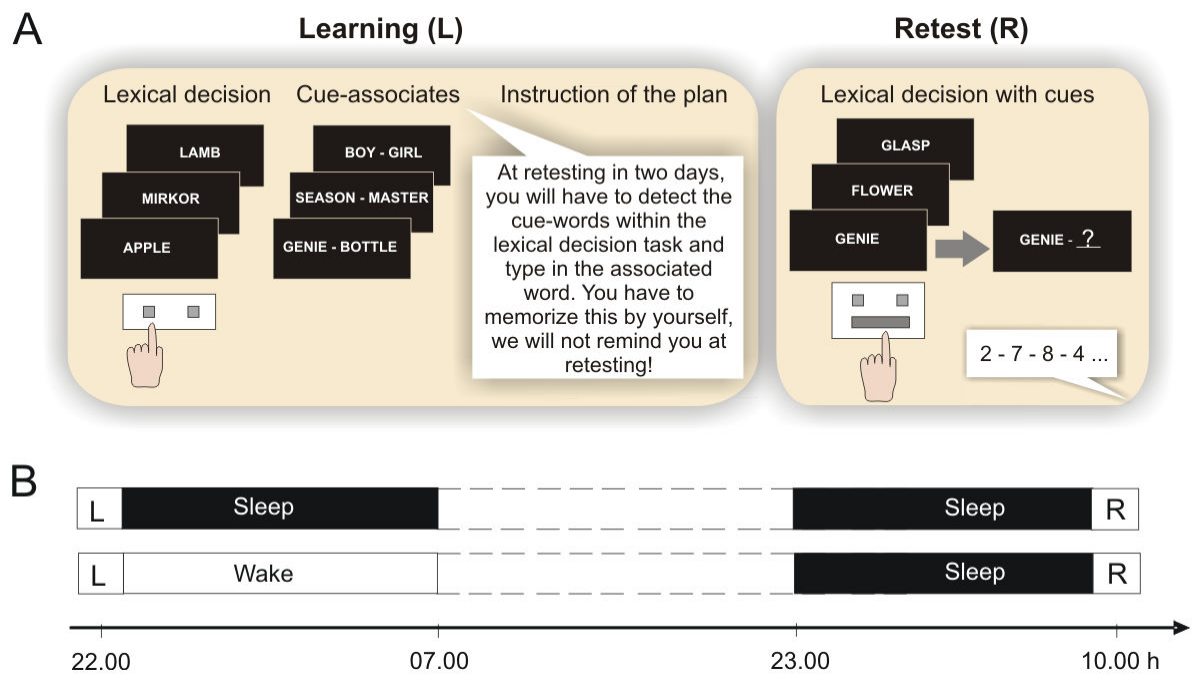
Prospective memory task and experimental design. (A) During learning, subjects practiced on the lexical decision task first, which required them to press one of two buttons indicating whether the presented word was a real word or not. Thereafter, subjects learned 20 cue–associate word pairs. For instruction of prospective memory, subjects were then told that at retesting on the lexical decision task two days later, some of the 20 cue words could occur within this task and if they detected a cue word they should press the 'space' bar and type in the respective associated word. Subjects were explicitly told that they need to memorize this instruction because the experimenter would not remind them of what to do at retesting. In order to manipulate available attentional resources, subjects performed at retesting in parallel a secondary task (monitoring spoken digits for two consecutive even digits) either during the first or second half of the lexical decision task. (B) Experimental design: Learning (L) took place in the evening (~22.00 h) followed by a night of sleep (sleep group) or wakefulness (wake group). Retrieval (R) was tested after another night of (recovery) sleep in both groups.

We applied a laboratory prospective memory task in which subjects were required to detect cues (i.e., specific cue words) and perform associated actions (i.e., recall associated second words) in an ongoing task (i.e., lexical decision task; Figure 1A). In the initial learning session, subjects first practiced the lexical decision task (serving as ongoing task later) without any prospective memory instructions. Subjects were presented in a random sequence with 100 word stimuli, half of which were existing German words. The other half were 'non-words' that were derived from German words by substituting one consonant [31,32]. Subjects were instructed to press as fast and as accurately as possible the right key (on a keyboard) for correct words and the left key for non-words (with the respective index finger). 

After practice on the lexical decision task, subjects learned 20 cue–associate word pairs for the subsequent prospective memory task. Half of the word pairs were semantically related, e.g. *Genie – Bottle*, half were semantically unrelated, e.g. *Season – Master*. Subjects learned the cue words first separately from the associated words. Cue words were presented successively for 5 sec each with 1-sec breaks in between. After presentation of all cue words, subjects recalled the words in a free recall test. Presentation and free recall was repeated to a criterion of 90% (i.e., 18) correctly recalled cue words to ensure that all subjects would perfectly recognize the cue words in a recognition test and prospective memory retrieval would not depend on how well cue words were learned. The 90% criterion in the free recall test was chosen based on pilot studies indicating that this criterion produced practically perfect performance on the word recognition test. After learning of cue words, subjects learned the respective associated words for each cue word. Word pairs were presented successively for 5 sec each with a 1-sec break in between. For each word pair, the cue word was presented on the left side of the screen and the associated word on the right side of the screen. After all word pairs had been presented once, a cued recall test followed in which subjects upon presentation of each cue word were required to press first the ‘space’ bar, then a field opened on the screen where they should type in the corresponding associated word. After pressing ’enter’, the next cue word appeared without feedback about the correctness of the previous response. Presentation of the word pairs and cued recall was repeated to a criterion of 60% (i.e., 12) correctly recalled associated words. The 60 % criterion was chosen based on previous studies indicating maximal effects of sleep on consolidation of word pair memories at this criterion [33]. 

The prospective memory instruction was given after the learning phase. Subjects were informed that, apart from testing their lexical discrimination abilities, we were also interested in their ability to remember to do something in the future. For this purpose, some of the cue words they had just learned would occasionally appear within the lexical decision task when they would be retested on this task two days later. When they detected a cue word at this retest they should press the ’space’ bar and then a field would open where they should type in the associated word, confirm with ’enter’ and continue with the task. Subjects had to repeat this instruction in their own words to ensure full understanding. They were explicitly instructed to memorize this instruction because at the retest session the experimenter would not remind them of what to do.

In the retest session, subjects performed the lexical decision task without being reminded of the instructed intention. The lexical decision task during retesting contained 390 word stimuli, i.e., 185 real words, 185 non-words and the 20 learned cue words. Cue words were presented every 16^th^ to 20^th^ word (mean: 18^th^). A break was introduced after half of the words had been presented. In order to test whether subjects used the resource-demanding monitoring strategy or the resource-independent spontaneous-associative strategy, we directly manipulated available attentional resources: during one of the halves of the task (balanced across subjects), subjects performed in parallel an auditory attention task in which spoken digits were presented via loudspeakers at a rate of one digit every two seconds. The subjects were required to monitor the spoken digits and press a separate key whenever two even digits occurred consecutively. 

### Design and procedure

Subjects were randomly assigned to the sleep group (*n* = 17) or the wake group (*n* = 18; Figure 1B). All subjects reported to the laboratory at 21.00 h, filled in questionnaires, and in the sleep condition electrodes were attached for standard polysomnographic recordings, including electroencephalogram (at sites C3 and C4), electrooculogram and electromyogram. Polysomnographic recordings were visually scored offline according to standard criteria as wake, sleep stages S1, S2, SWS (combining sleep stages S3 and S4), and REM (rapid eye movement) sleep [34]. The initial learning session took place between 22.00 and 22.45 h in both groups. In the sleep group, subjects were allowed to sleep between 23.00 h (lights off) and 07.00 h (awakening). Subjects in the wake group stayed awake throughout the night under supervision of an experimenter, spending the time with reading, watching TV or playing simple games. Subjects of both groups left the laboratory in the next morning. After spending the day awake and another night of sleep at home, allowing subjects in the waking condition to recover from their initial sleep loss, they returned to the laboratory for the retest session at ~10.00 h. Subjects kept record of their activities and their bedtime and wake-up time on the night at home. 

### Control variables

In the end of the retest session, memory for the cue words was tested in a recognition test. The 20 cue words were presented randomly mixed with 40 distractor words (not presented before) and subjects had to indicate for each word if it was a cue word or new. Following the recognition test, memory for the associate words was tested in a cued recall procedure. All of the 20 cue words were presented again and for each cue word subjects had to recall the associated word.

To control for general alertness and vigilance, all subjects performed on a vigilance task before learning and after retrieval testing. In this task, a dot randomly appeared at the left or right side of a computer screen every 2–10 seconds for about 10 minutes and participants had to respond as quickly as possible by pressing the corresponding left or right button. Subjects additionally rated their subjective sleepiness on the Stanford Sleepiness Scale before learning and after retrieval testing, ranging from 1 (“feeling active, vital, alert, or wide awake”) to 7 (“no longer fighting sleep, sleep onset soon; having dream-like thoughts”) [35].

### Statistical analysis

Three measures of prospective memory performance were obtained at retesting: (i) whether or not subjects remembered the intention at all, i.e., detected at least one cue word in the lexical decision task (reflecting overall prospective memory), (ii) the number of cue words detected (reflecting the prospective component), and (iii) the number of associated words remembered relative to the number of cue words detected (reflecting the retrospective component). The number of cue words detected and associated words remembered was further analyzed according to whether or not subjects’ attentional resources were reduced, i.e., with or without the auditory attention task to be performed in parallel, and whether cues and associated words were semantically related or unrelated. The number of subjects who remembered to detect the cue words in the lexical decision task (i.e., the overall prospective memory measure) was analyzed using χ^2^-tests. All other variables were analyzed using analyses of variance (ANOVA) and planned post-hoc *t*-tests. Additionally, non-parametric post-hoc tests (i.e., Mann-Whitney-U-Test and Wilcoxon-Test) were used when deviations from the normal distribution occurred due to positive skewness in the data, which was the case for the number of cues words detected and the final cue recognition test. Level of significance was set to *P* = 0.05. Greenhouse-Geisser correction for degrees of freedom was applied where appropriate.

## Results

### Prospective memory task performance

Initial learning performance of cue words and associated words was well comparable between sleep and wake subjects. Subjects in the sleep and wake group remembered 18.59 ± 0.17 and 18.83 ± 0.19 cue words in the criterion learning trial, *t*(33) = 0.97, *p* = 0.34, and needed on average 2.18 ± 0.25 and 2.61 ± 0.28 trials to reach the criterion, *t*(33) = 1.16, *p* = 0.26. Recall of associated words in the cued recall was on average 16.71 ± 0.68 and 15.11 ± 0.63, *t*(33) = -1.72, *p* = 0.09, after a mean of 1.29 ± 0.11 and 1.22 ± 0.11 learning trials, *t*(33) = -0.47, *p* = 0.64, in the sleep and wake group, respectively. 

**Figure 2:**
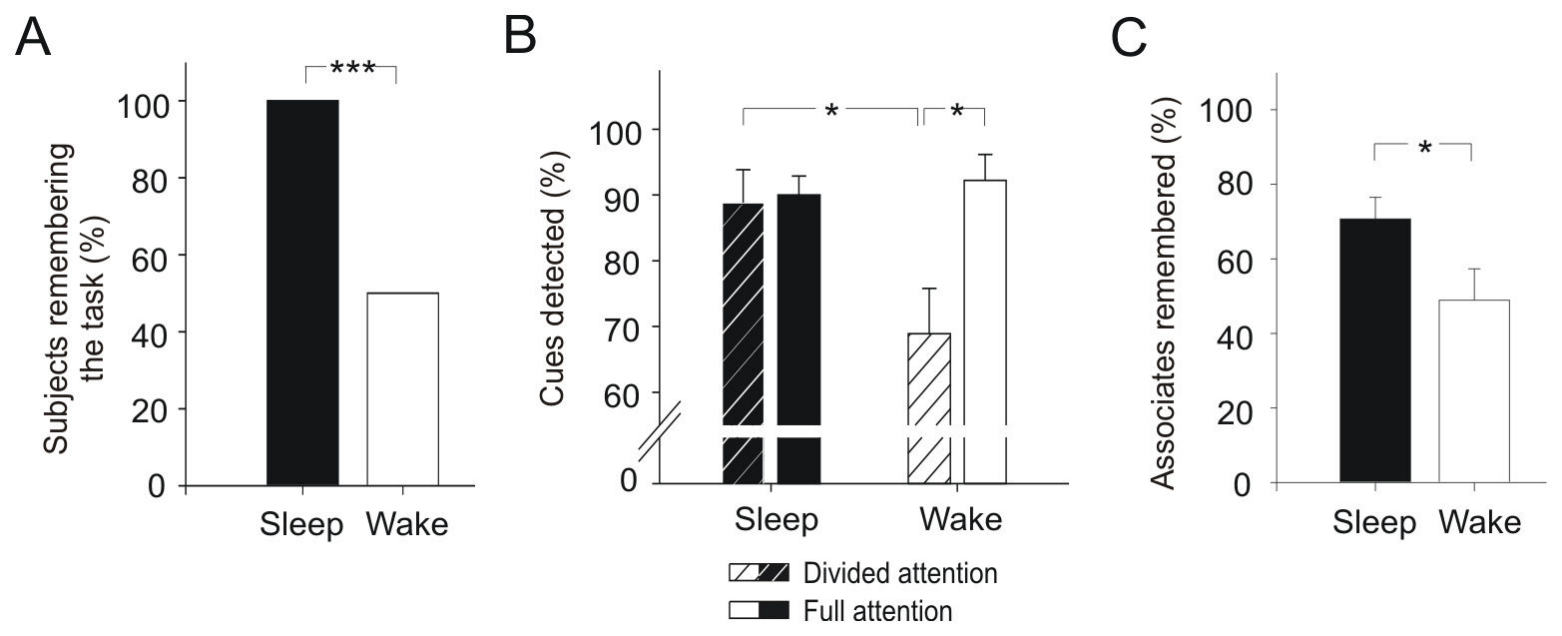
Prospective memory after sleep and wakefulness. (A) A significantly greater percentage of subjects who slept in the night after prospective memory instruction (sleep group) compared to subjects who stayed awake (wake group) retrieved the intention at a retest two days later, i.e., detected at least one cue in the lexical decision task. (B) The percentage of cue-words detected (a measure of the prospective component) differed in sleep and wake groups depending on whether subjects paid full attention to the task or engaged in parallel in a secondary (auditory attention) task. Subjects who slept after instruction of prospective memory were completely unaffected by divided attention, whereas wake subjects were strongly impaired in cue detection specifically during divided attention conditions. (C) The percentage of recalled word associates relative to the number of cues detected (measuring the retrospective component) was higher in sleep compared to wake subjects. Means ± SEM are shown. * *p* < 0.05, *** *p* < 0.001.

Regarding overall prospective memory performance at retesting, all subjects (100%) who slept after the prospective memory instruction remembered the intention, i.e., they detected at least one cue word within the lexical decision task, whereas in the wake condition only half of the subjects (50%) remembered to do so, χ^2^(1, *N* = 35) = 11.44, *p* = 0.001, d = 1.39 (Figure 2A). Thus, sleep after forming the intention enhanced the ability to implement the intention at the appropriate time two days later. 

Analyses of the prospective component of prospective memory, i.e., the number of detected cue words (restricted to the subjects remembering the intention, i.e., detecting at least one cue word), indicated that sleep facilitated cue detection particularly under divided attention conditions. Subjects who slept after learning remained completely unaffected by the secondary auditory attention task, detecting 90.00 ± 2.87% of the cue words without the secondary task and 88.82 ± 5.02% when the secondary task was performed in parallel, *Z* = -0.20, *p* > 0.80 (Figure 2B). Wake subjects, on the other hand, were markedly impaired in cue detection with reduced attentional resources available, detecting 92.22 ± 3.94% of the cues in the absence of the secondary task, but only 68.89 ± 6.89% when the secondary task was performed in parallel, *Z* = -2.24, *p* = 0.025, d = 1.10 (‘sleep/wake’ x ‘with/without secondary task’ interaction: *F*(1, 24) = 4.89, *p* = 0.037, η_*p*_^2^ = 0.17). Cue detection was not affected by whether cues and associated words were semantically related or unrelated (all *p* > 0.10 for main effect and interactions with ‘semantic relatedness’, see also Figure S1). 

Analyses of the retrospective component of prospective memory revealed that, relative to the number of cues detected, sleep subjects remembered 70.75 ± 5.77% of the associated words whereas wake subjects remembered only 48.92 ± 8.41%, *F*(1, 23) = 4.79, *p* = 0.039, η_*p*_^2^ = 0.17 (Figure 2C; note that this analysis excluded one of the wake subjects who did not detect any cue during divided attention). The difference between sleep and wake conditions did not depend on the presence or absence of the secondary auditory attention task (‘sleep/wake’ x ‘with/without secondary task’ interaction: *F*(1, 23) = 0.10, *p* = 0.75; ‘with/without secondary task’ main effect: *F*(1, 23) = 2.00, *p* = 0.17). Subjects overall remembered more semantically related than unrelated associated words, *F*(1, 23) = 38.88, *p* < 0.001, but the effect of sleep on memory for associated words was not affected by semantic relatedness (all *p* ≥ 0.10 for interactions with ‘semantic relatedness’, see also Figure S1). Interestingly, the percentage of recalled associated words significantly correlated with the number of cue words detected, but only during high attentional demands condition (i.e., with the secondary task performed in parallel), *r* = 0.48, *p* = 0.015 (versus *r* = ‑0.17, *p* > 0.40, without secondary task). This pattern of correlations suggests that recall of retrospective memories is related to intentional aspects inherent to the brain representation of these memories.

### Lexical decision task performance

Sleep and wake subjects did not differ in lexical decision performance at learning (mean reaction time, 1274 ± 83 vs. 1384 ± 117 ms, *p* > 0.40; error rate, 3.3 ± 0.5 vs. 4.3 ± 0.7%, *p* > 0.25) or retesting (averaged across trials with and without the secondary task: reaction time, 1523 ± 66 vs. 1575 ± 104 ms, *p* > 0.60; error rate: 3.8 ± 0.4 vs. 4.4 ± 0.5%, *p* > 0.30). In the retest session, performing the secondary auditory attention task in parallel slowed down reaction times for lexical decisions in both sleep subjects (1301 ± 94 vs. 1750 ± 94 ms) and wake subjects (1394 ± 92 vs. 1761 ± 92 ms; *p* < 0.001 for main effect ‘with/without secondary task’, *p* > 60 for ‘sleep/wake’ main effect, *p* > 0.30 for ‘sleep/wake’ x ‘with/without secondary task’), and increased error rates (sleep: 3.3 ± 0.5 vs. 4.3 ± 0.5%, wake: 3.5 ± 0.5 vs. 5.3%; *p* < 0.001 for main effect ‘with/without secondary task’, *p* > 0.30 for main effect ‘sleep/wake’, *p* > 0.25 for ‘sleep/wake’ x ‘with/without secondary task’). The slowing of reaction times was comparable in subjects who remembered to execute the intention, i.e., to detect the cue words (1363 ± 77 vs.1798 ± 79 ms) and those who did not (1310 ± 131 vs. 1634 ± 127 ms; *p* < 0.001 for main effect ‘with/without secondary task’, *p* > 0.40 for main effect ‘remembered/not remembered’, *p* > 0.20 for the interaction ‘with/ without secondary task’ x ‘remembered/not remembered’), confirming that the secondary auditory attention task actually put a high load on cognitive resources in all subjects independent of whether they remembered or forgot the intention. 

With reference to performance at learning, subjects who remembered the intention of detecting cue words showed a distinct slowing of reaction times for lexical decisions at retesting (by 266 ± 37 ms, *p* < 0.001), which was independent of the presence of the secondary task (*p* > 0.20) and comparable between the sleep and wake condition (*p* > 0.50). By contrast, there was no significant slowing of reaction times in the subjects who forgot the experimental intention (84 ± 85 ms, *p* > 0.35 [note that these were only wake subjects since all sleep subjects remembered to perform the task]; *p* = 0.028, for comparison of subjects who remembered the task and those who did not), supporting the view that subjects who did not detect a single cue word had indeed completely forgotten the intention. 

### Control variables

The final cue recognition test at the end of retesting was employed to exclude that reduced detection of cue words in the wake condition resulted simply from an impaired (retrospective) memory for the cue words themselves rather than the associations between cue words and associated words. Subjects in the sleep and wake condition recognized all the cue words perfectly well (recognition accuracy: sleep 99.15 ± 0.34%, wake 99.41 ± 0.20%, *P* > 0.80). The cued recall test of associated words further confirmed that sleep improved retrospective memory. In the sleep group subjects remembered 73.24 ± 5.23% of the associated words whereas wake subjects remembered only 51.94 ± 5.17%, *F*(1, 33) = 8.22, *p* = 0.007, η_*p*_^2^ = 0.22. When calculating recalled associated words relative to recall performance at learning (with learning set to 100%), subjects remembered 86.56 ± 5.03% and 68.23 ± 4.89% in the sleep and wake groups, respectively, *F*(1, 33) = 6.87, *p* = 0.013, η_*p*_^2^ = 0.17.

Sleep and wake subjects were further comparable in performance on the vigilance task and reported sleepiness during learning and retrieval (all *p* > 0.14, Table 1), excluding that differences between sleep and wake subjects in prospective memory performance were due to confounds by general changes in alertness. Subjects in the sleep group also displayed normal sleep patterns during the night following prospective memory instructions. Subjects slept on average 414.3 ± 14.2 minutes, including 18.9 ± 5.9 min of time awake after sleep onset, 19.7 ± 2.6 min of S1, 221.0 ± 10.4 min of S2, 75.1 ± 4.9 min of SWS, and 75.6 ± 5.6 min of REM sleep. None of the sleep variables was significantly correlated with any of the memory measures, neither with prospective memory performance (i.e. cue detection and remembered associates, all *p* > 0.15) nor with the final memory test (i.e. cue recognition and cued recall of associates, all *p* > 0.05). 

**Table 1:** Sleepiness and vigilance performance.

	**Sleepiness**		**Reaction times (ms)**		**Error rate (%)**	
	**Learning**	**Retest**	**Learning**	**Retest**	**Learning**	**Retest**
Sleep	2.35 ± 0.26	2.18 ± 0.21	318.05 ± 4.81	327.76 ± 8.31	5.13 ± 1.02	2.94 ± 0.75
Wake	2.17 ± 0.20	2.50 ± 0.22	330.75 ± 6.94	332.63 ± 6.93	7.36 ± 1.17	4.51 ± 0.80

Subjective sleepiness (Stanford Sleepiness Scale) and vigilance performance (reaction times in ms and error rates in % of all trials) during learning and retesting. There were no significant differences between the sleep and wake group. Means ± SEM are shown.

## Discussion

We found that a period of sleep following prospective memory instruction generally improved the ability to implement the intended behavior at the appropriate time after two days. Sleep not only enhanced the overall ability to remember the intention, but benefited both the prospective component of prospective memory to remember *that* something has to be done and the retrospective component of prospective memory to remember what has to be done. The prospective component thereby specifically benefited from sleep under conditions of reduced attentional resources, suggesting that sleep supported the consolidation of prospective memories in an associative memory network favoring spontaneous-associative retrieval processes. 

These results confirm and critically extend findings from previous studies on the beneficial role of sleep for prospective memory [29,30]. In the study by Scullin and McDaniel [30], for example, prospective memory performance was shown to be augmented after a 12 hour delay including one night of sleep compared to a 12 hour period of wakefulness. Importantly, relative to a control group that never encoded the prospective memory task, subjects who were allowed to sleep after prospective memory encoding did not show any costs in performance of the ongoing task, indicating that prospective memory was supported by spontaneous retrieval processes. Furthermore, the improvement in prospective memory occurred specifically in the context that was temporally paired with the prospective memory instruction, i.e., in the ongoing task that was performed immediately before the instruction. Together, these findings are suggestive of a memory-specific effect of sleep with sleep improving the consolidation of the association between the intention and the temporally paired context, thus facilitating the spontaneous retrieval and execution of the intended action. 

Our present data corroborate these conclusions by showing that sleep after prospective memory instructions supported memory for the prospective component specifically under conditions of high attentional demands. Subjects who stayed awake after instruction of the intention were impaired in the ability to detect relevant cues in the ongoing task when their attentional resources were reduced due to performance on a resource-demanding secondary task. This finding is in line with previous studies showing impaired prospective remembering under divided attention conditions [36,37]. Performance on a secondary task has been proposed to impair executive control and especially monitoring processes necessary to successfully detect prospective memory cues in an absorbing ongoing task [38]. Other studies, however, found no impairment of prospective memory performance by divided attention, suggesting that in these conditions subjects were able to recruit to a larger extent on resource-independent spontaneous retrieval processes [39]. It has been recently proposed that prospective remembering relies on the dynamic interplay of both attention-based monitoring processes and spontaneous-associative retrieval [17,18]. Which of the two processes prevails in a certain prospective memory task thereby depends on characteristics of the specific situation and prospective memory task, such as time interval, contextual cues, and prospective memory cue encounter [12,17,18]. Our present results indicate that the degree to which monitoring and spontaneous retrieval contribute to prospective remembering also depends on whether the period following encoding of the intention is filled with sleep or wakefulness. Upon first encounter of the context in which the intention had to be executed, subjects likely relied on spontaneous-associative retrieval to detect prospective memory cues because testing took place after 2 days and it seems unlikely that subjects engaged in continuous attention-demanding monitoring over a period of 36 hours [17]. This reliance on spontaneous retrieval resulted in better memory of the intention in subjects who slept after intention encoding: all sleep subjects remembered to detect the cues, whereas half of the wake group had forgotten the intention. Once subjects retrieved the intention spontaneously, both sleep and wake subjects relied at least partly on attentional monitoring resources as indicated by increased ongoing task costs when prospective memory cues were included in the ongoing task. It is further likely that all subjects used monitoring to some extent as we used a high number of prospective memory cues in the present study (i.e. 20) and it is known that monitoring increases with the number of different prospective memory cues [16]. Interestingly though, subjects who were allowed to sleep after encoding of the intention were able to recruit additional spontaneous-associative processes to support prospective memory performance under conditions of reduced attentional resources. While after wakefulness, subjects’ performance was impaired by divided attention, sleep subjects were completely unaffected by reduced attention resources. Thus, when prospective memory encoding was followed by a period of sleep, intention execution became partly independent of attentional resources, suggesting that in this case sleep strengthened the association between cues and associated actions, which boosted prospective memory performance via additional resource-independent spontaneous retrieval processes. 

This pattern of results indicates that sleep might facilitate the storage of intentions in an associative memory network. Specifically, we suggest that the process of sleep-dependent consolidation strengthens the connection between the prospective memory cue and the intended action. Sleep has been shown to strongly benefit the consolidation of associative memories like word paired-associates or card-pairs in an object-location task [40–43]. Cue-based prospective memory can be considered a kind of associative memory as the intended action becomes associated with a specific cue to signal that the intended action can be executed. By strengthening the cue–action associations sleep may provoke that intentions come to mind spontaneously once relevant cues are encountered, thus favoring a spontaneous retrieval of the intended action. Functionally, sleep can thereby release the cognitive system from persistent attention-demanding search for cues to execute intentions. Such a mechanism is highly adaptive in everyday life where we are busily engaged in all kinds of activities while bearing in mind our lasting intentions.

Apart from its beneficial effect on the prospective component, sleep also significantly improved retention of the retrospective component of prospective memory. Once subjects detected a cue word within the ongoing task, they were more likely to recall the associated word when they had been sleeping after instruction of prospective memory. Findings of the post-experimental cued recall test, assessing memory of the associated words when prompting subjects with the cues, confirmed a beneficial effect of sleep on memory for the retrospective component. Interestingly, the sleep-associated facilitation of the prospective component and the retrospective component were not independent. The strength of the retrospective component (i.e., number of recalled associated words) correlated significantly with the efficiency of the prospective component (i.e., cue detection). The fact that this correlation was selectively observed under conditions of divided attention, i.e., when subjects were more likely to rely on spontaneous-reflexive retrieval of intentions, suggests that sleep might indeed facilitate the consolidation of the association between the cue and the associated word favoring both cue detection and recall of the associated behavior. 

Whereas the prospective component to detect the cues within the ongoing task in wake subjects depended on the amount of attentional resources available, the retrospective component of recalling the associated words was independent of full or divided attention conditions. It can be assumed that the retrospective component of prospective memory closely resembles ‘normal’ retrospective memory since once the prospective memory cue is detected, recall of the associated intended action is basically similar to the process of prompted retrospective memory search [12,44]. Some previous studies suggest that retrieval of retrospective memory can be impaired when attentional resources are reduced due to concurrent activity on a secondary resource-demanding task [45], whereas others found only small or no reductions of memory retrieval under divided attention [46–48]. Recent evidence indicates that the distracting effect of divided attention on retrospective memory retrieval is process-specific. Memory retrieval is impaired by divided attention only when the memory task and the distractor task compete for resources in the same representational system (e.g., verbal) but not if both tasks recruit on different systems (e.g., verbal and numerical) [49–51]. Thus, our findings of the retrospective component being not impaired by divided attention conditions is in line with these studies considering that in the present study we applied a digit-monitoring task as distractor task and the retrospective component basically resembled a word cued recall test with both of these tasks recruiting different underlying processes.

There is a long-standing debate whether intentions over longer delays are upheld by sustained levels of activation or whether they become stored in a memory network [4,12,52]. Although our study cannot resolve this debate, the finding of a facilitating effect of sleep on prospective memory particularly under conditions of reduced attentional resources provides first hints that sleep might support the storage of intentions in the memory network thereby easing their later spontaneous retrieval. It can be speculated that the intentional aspect of a memory tags the representation for a facilitated access to sleep-dependent consolidation. We assume that consolidation during sleep originates from reactivations of the representation that, aside from hippocampal networks [42,53], extend to prefrontal cortex regions [54], possibly accommodating specifically intentional aspects of the representation. Representation of prospective memories comprises anterior parts of medial prefrontal cortex [9,55,56] together with hippocampal regions [57–59], suggesting that a coordinated neuronal reactivation in these networks might underlie the consolidation of prospective memories during sleep. Specifically, these reactivations may couple prefrontal intentional aspects of a representation to its retrospective aspects residing in hippocampal circuits [60]. 

Although our findings are consistent with the idea that sleep facilitates the storage of intentional cue-action associations in the associative memory network, the observed effects could alternatively be explained by sleep subjects simply having quantitatively greater amounts of attentional resources available than wake subjects. Higher amounts of attentional resources in the sleep group could have been due to, for example, prolonged detrimental effects of sleep deprivation on executive functioning in the wake group. A recent study found that subjects were impaired in prospective memory performance after 25 hours of acute sleep deprivation [61]. However, we believe that it is unlikely that sleep deprivation affected prospective memory performance in the present study. First, we introduced a recovery night after sleep deprivation to give wake subjects the opportunity to recover from their sleep loss. Second, our control data confirmed that subjects in the sleep and wake group were comparable with regard to attentional functioning as indicated by comparable reaction times in the vigilance task. Finally, in the study by Grundgeiger and colleagues sleep deprived subjects detected less prospective memory cues both in a cognitively demanding and a less demanding task, suggesting that sleep deprivation affected cognitive processing globally independent of whether the prospective memory task can be solved primarily with spontaneous retrieval or attentional monitoring processes [61]. In the present study, though, subjects in the wake group were specifically impaired in cue detection under cognitively demanding conditions, i.e., with the secondary task to be performed in parallel, but not under less demanding conditions without the secondary task, indicating that sleep deprivation did not affect prospective memory performance in our study.

Together, our findings suggest that sleep supports successful prospective remembering via boosting the storage of intentions in the memory network thereby facilitating the use of spontaneous retrieval processes at the appropriate time. Which particular psychological and neurophysiological mechanisms underlie the representation of prospective memories in the memory network will be an issue of future studies. Since we here show that sleep is functionally implicated in the maintenance of prospective memories, one outstanding question centers around understanding the sleep-associated mechanisms involved in this process.

## Supporting Information

Figure S1 **Sleep, prospective memory and semantic relatedness of cue-associate pairs.** (A) The percentage of detected cue words did not depend on whether cue-associate pairs were semantically related or unrelated. For both types of pairs, sleep subjects were unaffected by divided attention, whereas wake subjects were impaired in cue detection specifically during divided attention conditions. (B) Independent of whether cue-associate pairs were semantically related or not, sleep subjects remembered more word associates than wake subjects, relative to the number of cues detected. Generally, all subjects remembered more semantically related than unrelated word associates. Means ± SEM are shown.
(TIF)
