# Supplementary figures and images for: Sleep Improves Prospective Remembering by Facilitating Spontaneous-Associative Retrieval Processes
Source: PLoS One. 2013 Oct 15;8(10):e77621. doi: 10.1371/journal.pone.0077621 (PMC3797070; doi:10.1371/journal.pone.0077621)

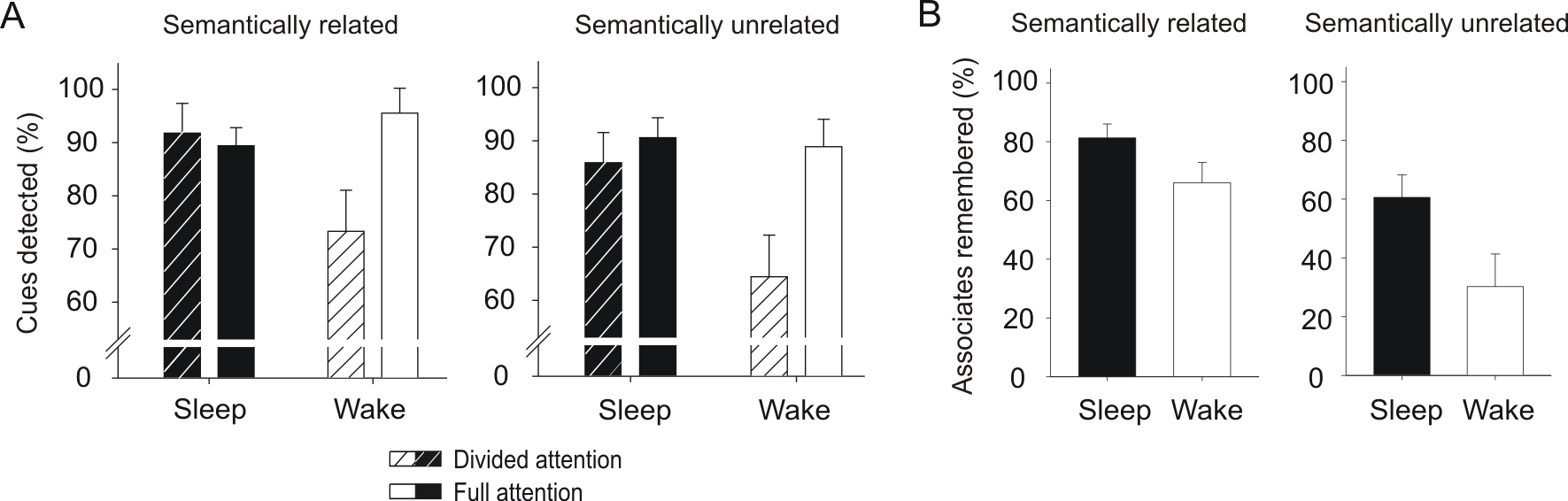

Supplement: Figure S1 — Sleep, prospective memory and semantic relatedness of cue-associate pairs. (A) The percentage of detected cue words did not depend on whether cue-associate pairs were semantically related or unrelated. For both types of pairs, sleep subjects were unaffected by divided attention, whereas wake subjects were impaired in cue detection specifically during divided attention conditions. (B) Independent of whether cue-associate pairs were semantically related or not, sleep subjects remembered more word associates than wake subjects, relative to the number of cues detected. Generally, all subjects remembered more semantically related than unrelated word associates. Means ± SEM are shown. (TIF) [file pone.0077621.s001.tif]
